# A purely visual adaptation to motion can differentiate between perceptual timing and interval timing

**DOI:** 10.1098/rspb.2023.0415

**Published:** 2023-06-14

**Authors:** Aurelio Bruno, Federico G. Segala, Daniel H. Baker

**Affiliations:** ^1^ Department of Psychology, University of York, Heslington, York YO10 5DD, UK; ^2^ School of Psychology and Vision Sciences, College of Life Sciences, University of Leicester, University Road, Leicester LE1 7RH, UK

**Keywords:** time perception, duration, adaptation, computational modelling, time scales

## Abstract

It is unclear whether our brain extracts and processes time information using a single-centralized mechanism or through a network of distributed mechanisms, which are specific for modality and time range. Visual adaptation has previously been used to investigate the mechanisms underlying time perception for millisecond intervals. Here, we investigated whether a well-known duration after-effect induced by motion adaptation in the sub-second range (referred to as ‘perceptual timing’) also occurs in the supra-second range (called ‘interval timing’), which is more accessible to cognitive control. Participants judged the relative duration of two intervals after spatially localized adaptation to drifting motion. Adaptation substantially compressed the apparent duration of a 600 ms stimulus in the adapted location, whereas it had a much weaker effect on a 1200 ms interval. Discrimination thresholds after adaptation improved slightly relative to baseline, implying that the duration effect cannot be ascribed to changes in attention or to noisier estimates. A novel computational model of duration perception can explain both these results and the bidirectional shifts of perceived duration after adaptation reported in other studies. We suggest that we can use adaptation to visual motion as a tool to investigate the mechanisms underlying time perception at different time scales.

## 1. Introduction

The ability to code and process time information in the millisecond range is essential for several everyday activities, ranging from action coordination to speech processing and recognition, to motion detection and processing, as well as more sophisticated behaviours like social interactions mediated by gaze. Although temporal processing on this scale is ‘probably the most sophisticated and complex form of temporal processing’ [1, p. 309], our knowledge of the underlying brain mechanisms remains quite poor. A similar lack of certainty affects the study of time perception in the order of seconds, where time is estimated in a more conscious fashion.

Some theories propose a single ‘internal clock’ mechanism, which would encode the duration of any interval regardless of the sensory modality of the embedded sensory stimulus, and of the time scale of the interval itself [2–4], simply by integrating the number of pulse-like signals generated by a pacemaker between the onset and offset of the considered interval. Empirical support for this model comes from the observations that increased arousal or attention induces duration overestimation, as they would speed up the clock [5,6], and that we cannot time two events simultaneously [7]. No biological substrate for this mechanism has been identified yet. Other theories suggest that time perception might be the product of a network of distributed mechanisms, which are modality-specific and contribute independently to time processing according to the time scale of the involved intervals [8]. Empirical support for this idea comes from the observations that many time perception biases like central tendency effects [9], rate after-effects [10] or duration after-effects [11] show little or no cross-modal transfer. Evidence exists of distributed mechanisms at both cortical and sub-cortical levels [12–14].

Time perception in the sub-second range is often referred to as ‘perceptual timing’ for being more tightly linked to perceptual processing and less susceptible to the influence of cognitive control, whereas, in the supra-second range, we refer to it as time estimation or ‘interval timing’, which is under a more direct control of higher cognitive functions like memory [15]. The idea that this distinction is reflected in different underlying mechanisms in our brain is supported by psychophysical [9,16], neuroimaging [17–20], neurophysiological [21] and pharmacological [22] studies.

Recently, adaptation has been used as a tool to study the mechanisms underlying time perception. Two different types of adaptation induce duration changes for sub-second intervals. First, adapting to several repetitions of an interval with a given duration resulted in repulsive duration after-effects, the magnitude of which depended on the temporal distance between the adaptor and test lengths [11,23]—but see Curran *et al*. [24]. This suggested the existence of duration-selective mechanisms with similar characteristics to those that process the spatial frequency or the orientation of a visual stimulus: adapting a given duration channel shifts the peak of the neuronal population response away from that duration, influencing the subsequent time judgements accordingly. A second type of adaptation does not address a specific duration channel and, nonetheless, induces a change in apparent duration. A purely visual adaptation to motion or flicker produces a substantial duration compression, which is temporal frequency-dependent, and it is limited to the adapting location [25–27]. The mechanisms involved in this type of adaptation are arguably not specific to the processing of duration, and they are most likely responsible for the processing of visual motion and temporal change.

While there is some evidence suggesting that the effect of duration adaptation might extend to supra-second intervals [28], we do not know yet whether adaptation to visual motion shows the same flexibility. If there was a dissociation between the two time scales, that would provide further evidence supporting the existence of time scale-dependent channels of time perception, and it would also shed some light on the brain sites where this type of adaptation takes place [29,30].

To address this issue, in this study, we measured perceived duration after space-specific adaptation to drifting motion for intervals centred around a sub-second duration (600 ms) or a supra-second duration (1200 ms), in separate sessions. Furthermore, we investigated whether adaptation interfered with the ability to discriminate changes in duration and whether duration changes depend on changes in duration discrimination after adaptation in the two time ranges. Finally, we developed a computational model of duration perception that was originally designed to explain size after-effects [31] and showed that it can account for both the duration compression after adaptation to motion and bidirectional repulsive duration after-effects [11,23].

## 2. Methods

### (a) Observers

Twenty observers (including two authors, all with normal or corrected-to-normal vision) participated in the experiment. All observers provided written informed consent prior to testing, and procedures were approved by the ethics committee of the Department of Psychology at the University of York (Ethics Application ID: 782).

### (b) Apparatus

Stimuli were displayed, in a dark room, on a gamma-corrected liquid crystal display (LCD) monitor (ASUS ROG Swift PG258Q), with a refresh rate of 240 Hz. We confirmed that our monitor indeed provided the correct timing of visual stimulus presentation by measuring different stimulus durations with a photodiode measurement circuit connected to an oscilloscope. The stimuli were generated in Matlab using the Psychophysics Toolbox extensions [32,33]. Stimuli were viewed from a distance of 57 cm. The head of participants was restrained with a chinrest.

### (c) Procedure

**Figure 1.**
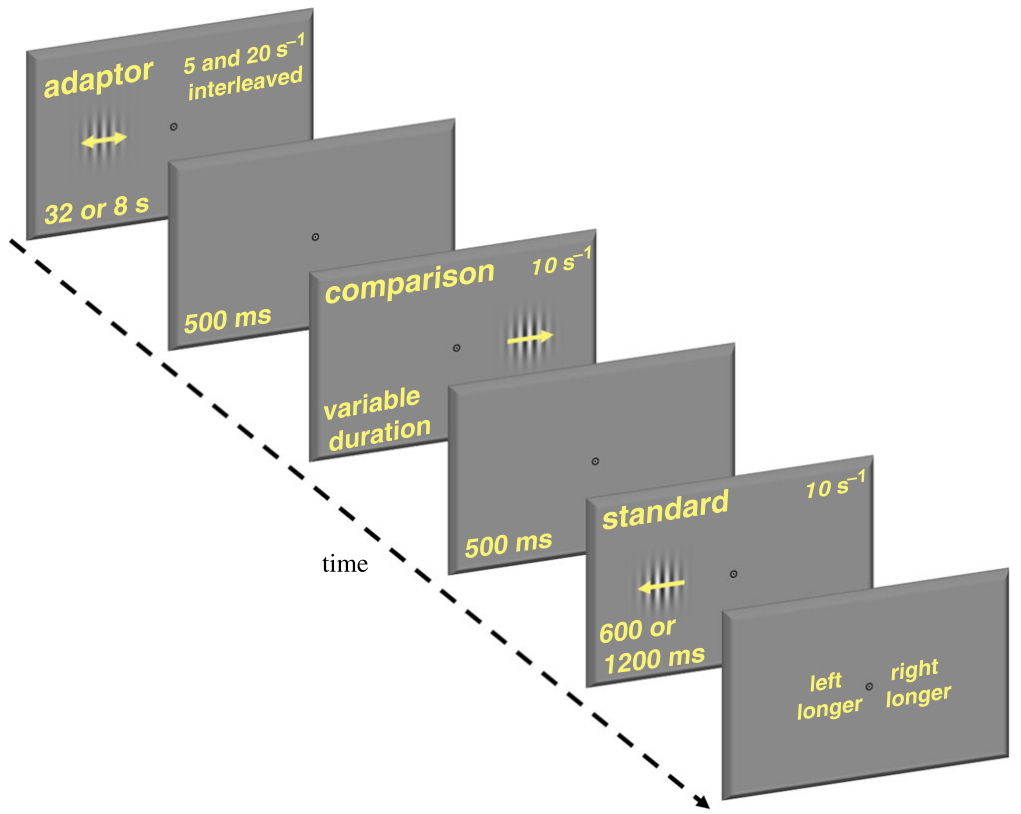
Schematic illustration of the procedure adopted in the adaptation conditions of the experiment. Participants adapted to an oscillating Gabor stimulus, which was displayed in a specific spatial location on the screen. Two test stimuli were subsequently displayed, one after the other: the standard, in the same location as the adaptor; the comparison, in an unadapted location. Participants reported which test had the longer duration.

All participants completed, in different sessions, an adaptation condition for a sub-second duration (600 ms), an adaptation condition for a supra-second duration (1200 ms) and two baseline conditions, one for each duration. The baseline conditions, without adaptation, were always completed before the adaptation conditions. The adaptation conditions were composed of an adaptation phase followed by a test phase (figure 1). Continuous fixation on a central spot was required for the whole duration of the experiment. All stimuli were drifting luminance-modulated Gabors (vertically oriented, spatial frequency: 1 cycle per degree; diameter of stimulus window: 5°; distance from the centre: 5°; standard deviation of the Gaussian spatial envelope: 0.83°). Michelson contrast was 50% for the adaptor and 80% for the tests to avoid reductions in apparent contrast in the tests after adaptation [34]. In the adaptation phase, participants saw an eccentric adaptor (5° to the left of the monitor centre), which reversed direction every 500 ms to avoid inducing a directional motion after-effect. The total adaptation time was 32 s for the first trial (with 8 s top-ups), divided into eight cycles of 4 s each (four cycles of 2 s each for the following trials). In each cycle, the adapting speed could be either 5 or 20° s^−1^ (the presentation order of the cycles was randomly interleaved on a trial-by-trial basis), so that the proportion of 5/20° s^−1^ adaptation was 50–50%, to minimize the effect of adaptation on the perceived speed of the tests [35–37]. When the adaptor disappeared, it was replaced by a blank screen of mean luminance for 500 ms, which preceded the test phase. In the test phase, two tests were sequentially displayed: one, the standard, in the same location as the adaptor, and the other, the comparison, in the opposite position relative to the fixation spot. The presentation order was randomized, and the two test intervals were separated by a 500 ms uniform mean luminance screen. Both tests drifted at 10° s^−1^ in opposite directions relative to each other. The standard had fixed duration across trials (either 600 or 1200 ms, in separate sessions), whereas the duration of the comparison varied in seven fractions (i.e. 1/3, 2/3, 5/6, 1, 7/6, 4/3, 5/3) of the standard duration to generate a psychometric function (for each comparison duration, we ran at least 20 repetitions). Participants were required to report which test had stayed on for the longer duration, by pressing one of two designated response buttons on a computer keyboard. We used custom Matlab code to fit cumulative Gaussian functions through the individual and mean data. The point of subjective equality (PSE, defined as the 50% point on the psychometric function) was our measure of perceived duration, whereas the just noticeable difference (JND, defined as half the difference between the 75 and the 25% points on the psychometric function) was our measure of duration discrimination.

### (d) Statistical analyses

We conducted all the statistical analyses using JASP software [38]. We report Bayes factors for *t*-test and correlational analyses (BF_10_) as well as for ANOVA analyses (BF_incl_). The latter were calculated as the sum of the posterior probabilities of all the models that contained the effect of interest divided by the sum of the posterior probabilities of all the models that did not contain the effect of interest.

## 3. Results

**Figure 2.**
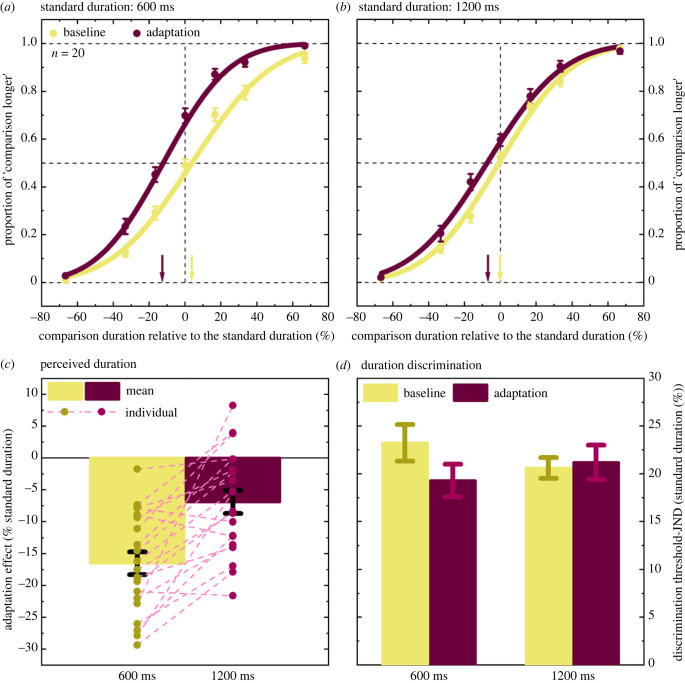
(*a*) Mean psychometric functions are plotted for the baseline (yellow circles and curve) and the adaptation conditions (dark red circles and curve), for 600 ms. The proportion of ‘comparison longer’ responses is plotted as a function of the comparison stimulus actual duration, expressed as a percentage change relative to the standard stimulus duration. The dark red and yellow arrows represent the point of subjective equality (PSE) for the adaptation and baseline conditions, respectively. Negative PSEs indicate duration compression. (*b*) The same as in (*a*) for 1200 ms. (*c*) The mean (columns) and individual (circles) adaptation effects, defined as the percentage difference between the PSEs in the adaptation and baseline conditions, are plotted for 600 ms (yellow) and 1200 ms (dark red). The dashed lines connect each participant's effects in the two standard duration conditions. (*d*) The just noticeable differences (JNDs) are plotted for the baseline and adaptation conditions and for 600 and 1200 ms. Error bars represent ± 1 s.e.m.

The psychometric functions (averaged across 20 participants) plotted in figure 2 describe participants' performance as a function of the difference in duration between the two test intervals, for the baseline and adaptation conditions and for the sub-second (600 ms, figure 2*a*) and supra-second (1200 ms, figure 2*b*) durations. Durations are expressed as percentages of the standard duration to facilitate the comparisons between physically different durations. The data represented in figure 2 are averaged across all participants; however, perceived duration estimates and duration discrimination estimates described below were derived from individual fits.

Overall, in the baseline conditions, our participants performed the task accurately: short comparison durations were infrequently judged as being longer than the standard duration, whereas for long comparison durations, the proportion of ‘longer’ responses was higher than chance. When standard and comparison intervals had the same duration (i.e. the ‘0″ duration in the plots’), participants' performance was at chance (even though, at 600 ms, the PSE was slightly higher than 0, PSE baseline 600 ms = + 3.74%, s.e.m. = 1.53, one-sample *t*-test, *t*_19_ = 2.45, *p* = 0.024, BF_10_ = 2.48). For the sub-second duration (figure 2*a*), adaptation induced a substantial leftward shift in the psychometric function relative to the baseline, which indicates a subjective compression of duration (PSE adaptation 600 ms = −12.75%, s.e.m. = 1.8, paired-samples *t*-test, *t*_19_ = 9.27, *p* < 0.0001, BF_10_ = 7.37 × 10^5^). This result confirms previous observations obtained in the millisecond range [8,25,27]. The effect of this type of adaptation for longer durations had not, to our knowledge, been systematically investigated before. For our supra-second duration (1200 ms, figure 2*b*), the duration compression observed after adaptation was substantially less pronounced, though still different from 0 (PSE baseline 1200 ms = −0.127, s.e.m. = 1.08; PSE adaptation 1200 ms = −7.02%, s.e.m. = 1.93; paired-samples *t*-test, *t*_19_ = 3.843, *p* = 0.001, BF_10_ = 33.73). Overall, adaptation induced a substantial duration underestimation relative to baseline (ANOVA repeated measures, main effect adaptation, *F*_1,19_ = 57.71, *p* < 0.0001, BF_incl_ = 2.21 × 10^9^), but the effect depended on the standard duration (interaction adaptation × standard duration, *F*_1,19_ = 27.92, *p* < 0.0001, BF_incl_ = 91.93).

To compare the relative magnitude of the duration changes for our sub- and supra-second intervals, in figure 2*c*, we plotted the adaptation effect, defined as the difference between adaptation and baseline estimates, expressed as percentage of the standard duration. The adaptation effect for 600 ms was more than twice as large as that for 1200 ms (adaptation effect 600 ms = −16.49%, s.e.m. = 1.78; adaptation effect 1200 ms = −6.89%, s.e.m. = 1.79; paired-samples *t*-test, *t*_19_ = −5.284, *p* < 0.0001, BF_10_ = 600.12). Each dashed line in figure 2*c* connects the individual adaptation effect for 600 ms with the corresponding adaptation effect for 1200 ms for the same participant. Seventeen of 20 participants showed a stronger adaptation-induced duration compression for the sub-second duration, and most participants showed a similar difference between the two durations (as highlighted by the fact that most of the dashed lines are almost parallel), indicating that the observed group effects captured the individual patterns of results well.

The duration compression described thus far could arguably be a consequence of a reduced sensitivity to duration differences (i.e. noisier estimates) after adaptation. If a change in the PSE was accompanied by a corresponding change in duration discrimination, it would be hard to claim that adaptation induced a specific effect on the subjective estimates of duration. Similarly, the difference in the magnitude of the adaptation effect between sub- and supra-second durations could potentially be due to different changes in duration discrimination in the two time scales. From figure 2, we can tell at a glance that this scenario is unlikely: adaptation does not seem to dramatically change the slopes of the psychometric functions. A statistical analysis confirmed this impression. In figure 2*d*, we plotted the JNDs, as a measure of duration discrimination threshold, for all conditions. Overall, duration discrimination improved slightly after adaptation (ANOVA repeated measures, main effect adaptation, *F*_1,19_ = 5.016, *p* = 0.037, BF_incl_ = 0.64). Even though there was weak evidence supporting an interaction between the adaptation and standard duration factors (*F*_1,19_ = 3.985, *p* = 0.06, BF_incl_ = 2.06), direct comparisons between adaptation and baseline conditions showed that the mean JND was moderately lower after adaptation for 600 ms (JND baseline 600 ms = 23.24%, s.e.m. = 1.92; JND adaptation 600 ms = 19.3%, s.e.m. = 1.71; paired-samples *t*-test, *t*_19_ = 2.892, *p* = 0.009, BF_10_ = 5.42) but not for 1200 ms (*t*_19_ = −0.43, *p* = 0.67, BF_10_ = 0.253).

In principle, there could still be a significant individual trend that linked changes in perceived duration to changes in duration discrimination. Group statistics cannot, in fact, completely exclude that a particular direction in the duration estimates (towards duration compression, for example) was systematically associated to noisier or better duration discrimination. To investigate this possibility, we plotted the individual PSEs against the individual JNDs, for both 600 and 1200 ms (electronic supplementary material, figure S1). The correlation between the two measures was strong only for the baseline condition at 600 ms (Pearson's *r* = 0.669, *p* = 0.001, BF_10_ = 35.4), indicating that the higher the duration discrimination thresholds were, the larger the overestimation of interval duration. We can therefore claim that duration changes after adaptation did not depend on changes in duration discrimination.

One might wonder whether the stronger adaptation effect observed for 600 ms relative to 1200 ms was in fact driven by differences already present in the baseline conditions. As we reported above, mean estimates for 600 ms were slightly higher than 0, indicating duration overestimation. Moreover, the amount of this overestimation was positively correlated with the duration discrimination thresholds (electronic supplementary material, figure S1*a*). To probe this alternative explanation, we re-analysed the data, excluding the three participants with the highest PSEs for the baseline condition at 600 ms. They all hugely overestimated the standard duration without adaptation (by 11.07, 15.73 and 21.73%, respectively), and their PSEs were substantially separated from those of the rest of the participants (electronic supplementary material, figure S1*a*). This exclusion was enough to reduce the average PSE to 1.54% (s.e.m. = 1.02), which was no longer higher than 0 (one-sample *t*-test, *t*_16_ = 1.52, *p* = 0.15, BF_10_ = 0.65). The correlation between the PSEs and the JNDs for the same condition also became weak (Pearson's *r* = 0.175, *p* = 0.5, BF_10_ = 0.369). However, the adaptation effect for 600 ms remained twice as large as for 1200 ms (mean adaptation effect 600 ms = −15.66%, s.e.m. = 2.03; mean adaptation effect 1200 ms = −6.97%, s.e.m. = 1.82; paired-samples *t*-test, *t*_16_ = −5.227, *p* < 0.0001, BF_10_ = 334), discarding the possibility that this difference was due to a tendency to overestimate duration without adaptation.

## 4. Computational modelling

Adaptation after-effects such as the tilt after-effect are typically modelled using a population of tuned units that tile the stimulus space [39]. Perception of a given stimulus is determined either by the mechanism with the greatest response or by a more sophisticated population read-out rule such as maximum-likelihood decoding [40]. Adapting to a particular stimulus shifts the peaks of the adjacent mechanisms away from the adaptor, producing a bidirectional repulsive after-effect. This general scheme was proposed by Heron *et al*. [11] to account for their bidirectional duration after-effects. However, their model is implemented only at the stage of tuned duration channels and lacks a front end that can process stimuli with arbitrary temporal properties (such as the drifting stimuli we use here).

**Figure 3.**
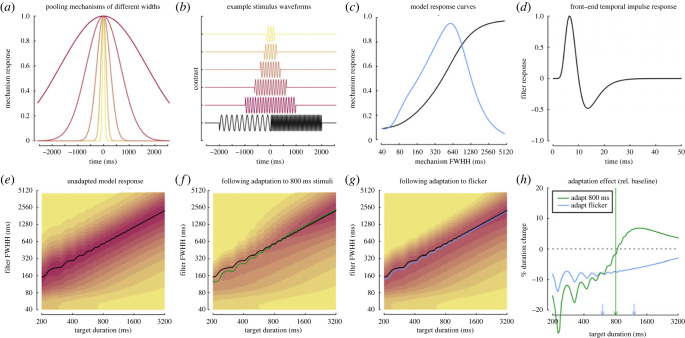
Summary of model components and behaviour. (*a*) Example pooling windows for five mechanisms that integrate over different durations. (*b*) Example stimulus waveforms for 10 Hz modulated targets of different durations, and the flickering 5 Hz/20 Hz adaptor (black). (*c*) Model responses to an 800 ms target, as a function of mechanism width. The black curve is the output of the non-linear transducer, and the blue curve is the derivative (obtained via subtraction between adjacent mechanisms). (*d*) We show the temporal impulse response function used as a front-end filter for the model. (*e*) The model response as a joint function of target duration and filter width, with the black curve showing the location of the peak response prior to adaptation. (*f,g*) The model response following adaptation to either an 800 ms 10 Hz target (*f*) or the 5 Hz/20 Hz flicker (*g*). Black curves in (*f*,*g*) are duplicated from (*e*), and the green and blue curves indicate the peaks following adaptation. (*h*) The predicted change in perceived duration after adapting to a fixed duration (800 ms) or a flickering adaptor. FWHH, full width at the half-height.

Our aim was to construct a model that could account for both the repulsive after-effects observed for a fixed-duration adaptor [11,23] and the duration compression effects we report here when using a drifting adaptor. To do this, we converted a model developed by Meese & Baker [31] to explain analogous after-effects for stimulus size (i.e. a 2° adaptor causes 1° targets to appear smaller and 4° targets to appear larger) for use in the time domain. A more extensive derivation of the model can be found in the electronic supplementary material. In brief, incoming stimuli are processed by a bank of mechanisms sensitive to different durations (figure 3*a*). The output of each mechanism is then subject to non-linear transduction, which involves divisive suppression from the largest mechanism (in the spatial domain this is surround suppression). Across the population of mechanisms, the response to a single stimulus (examples in figure 3*b*) is then a saturating sigmoidal function (black curve in figure 3*c*). To determine the peak response, the derivative is calculated by taking the difference between each mechanism and its neighbour (blue curve in figure 3*c*).

To handle arbitrary temporal waveforms, we added a ‘front end’ to the model, consisting of a temporally band-pass biphasic impulse response function (figure 3*d*). We did consider using multiple temporal filters, as suggested by Johnston [41–43]; however, these were not required to reproduce our effects. Other filters, such as a low-pass filter, behaved similarly for the conditions we consider. Adaptation is implemented by adjusting the gain of each mechanism in proportion to its response to the adapting stimulus. See the electronic supplementary material for a formal mathematical account and the online modelling code for implementation.

Figure 3*e* illustrates that, prior to adaptation, the model gives a duration read-out (black curve) that increases in proportion to stimulus duration. Note that the ‘wiggles' in the curve at brief durations are due to interactions between the phase of the sinusoidal carrier and the stimulus duration envelope. When the model is adapted to a single duration (800 ms), as in Heron *et al*. [11], perceived duration is shifted away from the adaptor duration in both directions (see the green curve in figure 3*f,h*). Adapting to flicker at 5 and 20 Hz (equivalent to a drifting stimulus from the perspective of a local one-dimensional filter) instead produces a unidirectional shift, whereby perceived durations of all target stimuli are reduced (blue curve in figure 3*g,h*). However, this effect is largest at shorter durations and reduces for longer durations, in qualitative agreement with our empirical results (figure 2*c*). The model architecture is therefore able to explain the main features of both repulsive and compressive after-effects within a single framework.

## 5. Discussion

We used a well-established adaptation paradigm [8,27] to investigate the effect of adaptation to visual motion on perceived duration and duration discrimination for both sub- and supra-second intervals. We found that adaptation perceptually compressed the duration of a 600 ms interval and that the magnitude of the effect relative to a baseline condition without adaptation corresponded to about 16% of the interval length. For a 1200 ms interval, we observed a much weaker duration compression: the adaptation effect amounted to about 7% of the interval length. Duration discrimination improved slightly after adaptation, especially for the sub-second duration. We did not observe any significant relationship between the changes in perceived duration after adaptation and the corresponding changes in duration discrimination.

The distinction between ‘perceptual timing’ and ‘interval timing’ for the processing of sub- and supra-second intervals, respectively, was mainly based on the absence (for the former) or presence (for the latter) of cognitive control. Perceptual timing would rely on more sensory and automatic processes, which arguably occur in early pre-cortical and cortical sensory sites, whereas interval timing would depend on more cognitive processes, which arguably occur in higher-order cortical areas. Recent systematic meta-analyses of neuroimaging studies confirmed these predictions. Even though there was a quite high degree of overlap between networks of areas involved in millisecond and second processing (mainly in the frontal lobes, where the supplementary motor area was almost always active in relation to timing tasks), sub-cortical areas, like the cerebellum and basal ganglia, were found to be more involved in sub-second tasks, whereas supra-second tasks required more cortical contributions, especially from the inferior parietal cortex [19,20]. Studies using transcranial magnetic stimulation reported disruptions of the performance in timing tasks for sub-second, but not supra-second intervals after stimulation to the cerebellum, and the opposite pattern when the stimulation occurred over the dorsolateral prefrontal cortex [44,45]. The difference we observed in this study between the effect of adaptation on sub-second relative to supra-second intervals might therefore suggest that this adaptation addressed more sub-cortical, rather than cortical components. This is consistent with previous observations that adapting to a temporal frequency above the flicker fusion threshold still induced duration compression [46]. Using electroencephalograms, Tonoyan *et al*. [47] looked at the electrophysiological signal in a duration task in the sub-second range, after adaptation to visual motion. Changes in the amplitude of the N200 component (which is thought to arise from area V5/MT), recorded in contralateral occipital electrodes, reflected changes in perceived duration after adaptation. They also found that changes in beta power after adaptation could predict duration compression. Taken together, these results suggest that the effect of motion adaptation on perceived duration in the sub-second range occurs locally and relatively early in the visual system.

The adaptation effect at 1200 ms interval was weak but did not completely disappear in our data. This could be because this duration is barely in the supra-second range, and the shortest two of the seven comparison durations we used were in the sub-second range. It is not clear where the boundary between sub- and supra-second processing can be traced [14]. The coefficient of variation, corresponding to the s.d. of a time estimate over the mean, measured in various timing tasks, increases more abruptly at approximately 1500 ms, suggesting a possible boundary [48,49]. We chose 1200 ms and not a longer duration to prevent our participants from using a counting strategy to estimate duration [50] and to keep the duration of our adaptation study to a manageable length. Therefore, at this stage, we can only speculate that, at longer supra-second durations (e.g. 1500 ms or longer), the effect of motion adaptation might become negligible.

The adaptation to a period of continuous visual drift we used here is different from the adaptation to several repetitions of a specific duration described by Heron *et al*. [11], which we could call ‘duration adaptation’. In our case, in fact, we were not adapting a putative duration channel but rather a channel sensitive to visual speed or temporal frequency. We will call it ‘motion adaptation’. We showed here that the effect of motion adaptation on duration perception is significantly weaker in the supra-second scale. On the contrary, Shima *et al*. [28] reported comparable effects after duration adaptation for the two time ranges and that adaptation transferred from one range to the other. Duration adaptation and motion adaptation were shown to differ across other dimensions as well. Duration adaptation in the visual domain, in fact, shows a very broad spatial tuning [51–53] and a strong interocular transfer [13], suggesting a late rather than early cortical site for this kind of adaptation to occur. Consistent with this prediction, neuronal activity in the inferior parietal lobule in response to a given sub-second duration was shown to be maximally reduced by recent exposure to the same duration and progressively less so when the difference in duration between the two repetitions increased [54]. Conversely, motion adaptation shows a very narrow spatial tuning [36] and no interocular transfer [37]—but see Burr *et al*. [25]—pointing to an early rather than late brain locus. However, we note that our computational model can reproduce both of these effects within a single framework, using a common mechanism of adaptation, and it can also predict the weaker effect of motion adaptation for longer durations. Our model was originally developed to account for repulsive size after-effects after adaptation [31]. In the space domain, adapting to a given size reduces the perceived size of a subsequently displayed smaller object, whereas it increases that of a larger object. Analogous effects also occur for spatial frequency [55], and there is an interaction between grating frequency and perceived object size [56]. In the time domain, a similar repulsive after-effect was shown after duration adaptation [11]. The motion adaptation effect on duration reported here bears some similarity with the compression of perceived spatial extent after adaptation to dynamic random dot texture [57,58]. They are both space-specific non-repulsive after-effects, and the latter does not depend on changes in perceived density, as the former is dissociable from changes in perceived speed. Arnold *et al*. [59] showed that adaptation to fast flicker enhances the acuity of spatial vision by mitigating the contribution of magnocellular neurons, which have poor spatial resolution. With their high temporal resolution, the involvement of magno cells in time perception has been often hypothesized [41,42,60].

Fornaciai *et al*. [61] reported that duration compression occurred only after adaptation to unidirectional translational motion and not after circular motion, radial motion or when the adaptor consisted of multiple patterns of translational motion moving in different directions. Here, our drifting adaptor reversed direction every 500 ms; therefore, our participants did not adapt to a single direction of motion. Nevertheless, duration compression was substantial. Also, previous studies [27,46] showed that adaptation to flickering patterns induced a duration compression comparable to that observed after drifting motion, suggesting that temporal frequency, not motion, is the key factor. Our model does not contain a directional component, and it assumes that both the adaptor and the tests are flickering, as flickering and drifting patterns are identical for a local one-dimensional filter.

Chronotopic maps with a topographical organization were found in several brain areas, ranging from sensory occipital cortices to motor frontal cortices [62,63]: neuronal populations in these areas show a maximal response to a preferred interval duration, whereas their activity is inhibited by non-preferred durations. The described timing selectivity seems to depend on the time scale, with a less defined spatial progression of the chronotopic maps for supra-second durations and with variable map sizes depending on the duration range. These maps capture well the characteristics of the ‘duration channels’, which would be affected by duration adaptation [11]. Even though chronotopic maps were found in visual areas that are involved in motion processing [62], it is less clear how these brain mechanisms can account for the effects of motion adaptation. The adaptation durations we used in this experiment (32 or 8 s) are longer than any of the test durations, and, therefore, the duration compression we observed might have resulted from adapting a duration channel, like those we have just described. However, Heron *et al*. showed that their duration channels are narrowly tuned around the preferred duration and the effect of adaptation tended to disappear when the difference between adapting and test durations was larger than 1.5 octaves. The same difference in our paradigm is substantially larger than that; therefore, we should not expect any effect of duration adaptation on our results. Following the same logic, our supra-second duration should have shown a bigger compression than the sub-second duration, as it is closer to the adapting duration, but this was not the case.

It has been recently shown that adaptation to a slow or a fast rate can induce duration overestimation and underestimation, respectively, of empty intervals defined by transient signals at onset and offset [64], suggesting that the observed after-effects do not depend on the interval content. On the contrary, adaptation to visual motion or flicker biases perceived duration even without changes in perceived onset and offset [26,27], implying that the content of an interval is taken into account when duration is processed, as hypothesized by some models [41,42,65]. The observation that intervals with equal length containing stimuli with different speed temporal profiles are perceived to have different durations [60,66–68] also points to a central role of interval content in duration perception. Comparing the actual and subjective durations of intervals containing stimuli drifting at a constant speed with that of intervals containing accelerating stimuli with the same average speed, in a functional magnetic resonance imaging (fMRI) study, Binetti *et al*. [12] observed two separate subsets of activated areas, one including early visual areas for objective durations and one including more anterior areas and the cerebellum for subjective durations. This is further evident that duration perception is achieved by a network of distributed mechanisms with different levels of involvement in the extraction and estimation of duration information from a sensory signal.

The bias in perceived duration after adaptation was not due to a reduced ability to discriminate between durations; on the contrary, duration discrimination thresholds lowered after adaptation (especially for 600 ms), indicating an enhanced sensitivity. This is in line with other vision studies that showed better discrimination after adaptation to contrast [69], orientation [70] or face identity [71]. Speed adaptation induces subsequent changes in perceived speed accompanied by an increase in speed discriminability [72,73], the same pattern we observed here for duration. We minimized the changes in apparent speed after adaptation using a ‘mixed’ adaptor, which alternated between two frequencies (5 and 20° s^−1^) known to induce opposite speed after-effects, which were shown to cancel each other [35–37]. Even under these conditions, the duration bias did not disappear, further implying that duration changes after adaptation are dissociable from changes in perceived speed. To our knowledge, no studies have reported differences in perceived speed between stimuli embedded in sub- versus supra-second intervals or a different effect of 5 and 20° s^−1^ adaptation on a 10° s^−1^ test embedded in a supra-second interval.

In conclusion, our results support the idea that the processing of duration information contained in a sensory signal is achieved by multiple mechanisms, which are independently recruited according to the duration range of the intervals to estimate. The advantage of having several different mechanisms, which might appear to represent time in a redundant fashion, is twofold: first, if necessary, they allow us to deal with duration information from multiple intervals simultaneously, and second, as we have different spatial brain maps to guide perception and action, we can benefit from having different mechanisms specialized for different purposes and functions. Adaptation to visual motion, for its well-defined spatial and temporal characteristics and for the robustness of the effects it induces on subjective duration, is an ideal tool to investigate the different contributions of cortical and sub-cortical components to the representation and experience of interval duration at different time scales.

## Data Availability

Experimental and analysis code, and raw and processed data presented in this manuscript are available at: https://osf.io/ps397/.
